# Effectiveness of antibacterial agents against cell-invading bacteria such as *Streptococcus pyogenes* and *Haemophilus influenzae*

**DOI:** 10.1186/s12866-021-02217-y

**Published:** 2021-05-14

**Authors:** Hiroyuki Iuchi, Junichiro Ohori, Satoshi Kiyama, Naoko Imuta, Junichiro Nishi, Yuichi Kurono, Masaru Yamashita

**Affiliations:** 1 Department of Otolaryngology, Head and Neck Surgery, Kagoshima University Graduate School of Medical and Dental Sciences, Kagoshima University, 8-35-1 Sakuragaoka, Kagoshima, 890-8544 Japan; 2 Department of Microbiology, Graduate School of Medical and Dental Sciences, Kagoshima University, 8-35-1 Sakuragaoka, Kagoshima, 890-8544 Japan

**Keywords:** Antibacterial agents, *Haemophilus influenzae*, *Streptococcus pyogenes*, Susceptibility, Garenoxacin, *emm* typing

## Abstract

**Background:**

Recurrent tonsillitis is one of the most common otolaryngological disorders caused by cell-invading bacteria, such as *Streptococcus pyogenes (S. pyogenes)* and *Haemophilus influenzae*. The aim of this study was to investigate the effect of antibacterial agents against cell-invading bacteria.

**Methods:**

The intracellular invasion of Detroit 562 cells by five strains of nontypeable *Haemophilus influenzae* (NTHi) and four strains of *S. pyogenes* was investigated. The antibacterial agents used were garenoxacin (GRNX), clarithromycin (CAM), amoxicillin (AMPC), cefditoren pivoxil (CDTR-PI), and levofloxacin (LVFX).

**Results:**

Both NTHi and *S. pyogenes* fully invaded Detroit 562 cells in 6 h and were less sensitive to CAM. GRNX, CAM, and LVFX were effective against bacteria invading the cells, but AMPC and CDTR-PI were not effective. GRNX was the most effective.

**Conclusion:**

GRNX was the most effective agent against bacteria invading cells.

## Background

Recurrent tonsillitis is one of the most common otolaryngological disorders [1]. The most frequent cause is viruses, and the second most frequent cause is bacteria, such as *Streptococcus pyogenes (S. pyogenes)*, *Staphylococcus aureus*, *Haemophilus influenzae*, and *Streptococcus pneumoniae* [2]. Among them, nontypeable *H. influenzae* (NTHi) and *S. pyogenes* invade the cells and escape from the action of antibacterial agents [1]. When tonsillitis is not cured by initial treatment, the current antibacterial agents must be replaced with agents that combat bacteria that have invaded the cells.

*H. influenzae* is a leading cause of acute and chronic otitis media, chronic sinusitis, and tonsillitis [3]. It is reported that in otitis media and chronic sinusitis, most strains of *H. influenzae* lack capsular polysaccharides and are referred to as NTHi and that *H. influenzae* frequently persist within dense biofilm communities that are thought to provide resistance to host clearance and bactericidal activity of some antibacterial agents [4]. *S. pyogenes* is an important human pathogen that can cause severe, life-threatening, invasive infections, such as soft tissue infection, sepsis, and streptococcal toxic shock syndrome [5]. *S. pyogenes* is generally an extracellular pathogen that can survive and persist within the host by the expression of a broad array of virulence functions directed to circumventing the host immune mechanisms [6]. It is believed that recurrent tonsillitis is caused by NTHi and *S. pyogenes* entering cells and escaping from the action of antibacterial agents.

International guidelines recommend penicillin as the first-choice antibiotic treatment for acute sore throat (suspected to be caused by *S. pyogenes*) [7]. However, a recent meta-analysis of clinical studies reported that cephem agents are more effective than penicillin agents [8] and are effective as short-term therapy [9]. Therefore, it has become necessary to reconsider the conventional treatment policy based on penicillin. In Japan, β-lactamase-negative ampicillin-resistant *H. influenzae* (BLNAR) is particularly common [10]. Therefore, tonsillitis that is not cured by initial treatment requires a change of antibacterial agents or the selection of antibacterial agents against bacteria that have invaded the cells.

Levofloxacin (LVFX), a broad-spectrum fluoroquinolone with potent activity against Gram-positive bacteria, is currently recommended to treat respiratory tract infections and pneumonia due to *S. pneumoniae*, one of the most important causative pathogens in community-acquired pneumonia (CAP). Similarly, garenoxacin (GRNX) is an oral des-fluoro (6)-quinolone with potent antimicrobial activity against common respiratory pathogens [11]. LVFX and GRNX show similar antimicrobial activities against Gram-negative bacteria. However, GRNX has higher antimicrobial activity than LVFX against Gram-positive bacteria, including staphylococci, streptococci, and pneumococci [12]. Additionally, GRNX has higher broad-spectrum antimicrobial activity against anaerobes than LVFX [13]. These data suggest that GRNX may be an attractive agent for the treatment of CAP.

Clarithromycin (CAM) exerts its antibacterial activity through its inhibitory effect on protein synthesis and is therefore effective against atypical pathogens such as *Mycoplasma pneumoniae* and *Chlamydia pneumoniae* that do not have cell walls [14]. It also has antibacterial activity against intracellular parasites such as *Legionella* and nontuberculous mycobacteria, reflecting its excellent transferability from tissues to cells [14].

The present study investigated the in vitro antibacterial activity of antibacterial agents against clinical strains of NTHi and *S. pyogenes* isolated in Japan.

## Results

### Bacterial invasion time

**Fig. 1:**
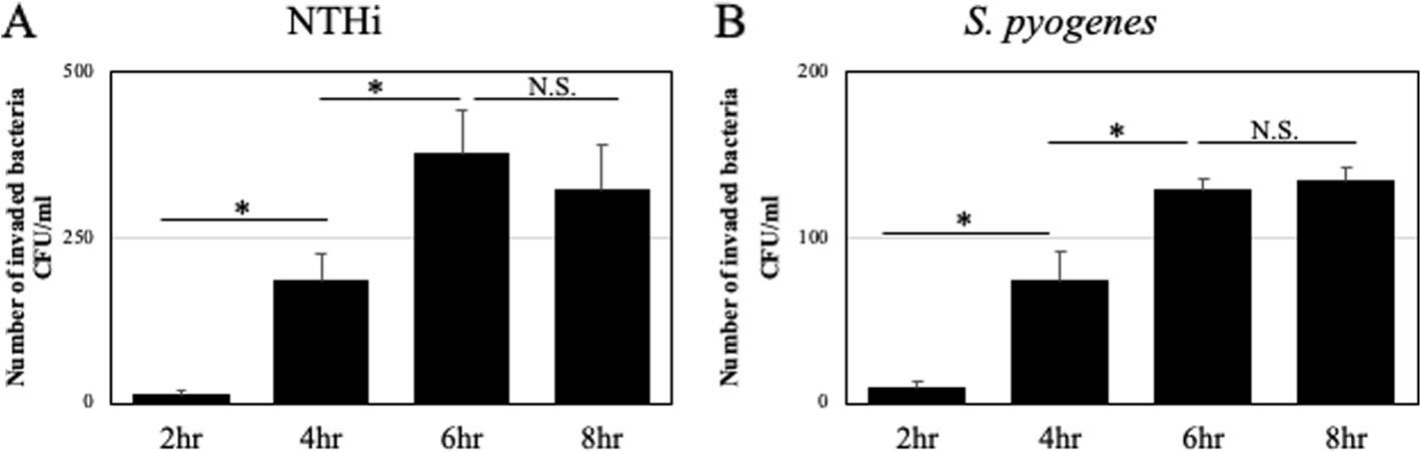
Bacterial invasion time. Nontypeable *Haemophilus influenzae* (NTHi) entered the cells 2 h after adhering to the cells (**a**). The maximum invasion was at 6 h (**a**). There were no differences between the numbers of bacteria invading the cells after 6 h and 8 h (**a**). Similar results were also observed with *Streptococcus pyogenes*, which invaded the cells 2 h after cell adhesion (**b**). The maximum invasion was at 6 h (**b**). No differences were found between the numbers of cell-invading bacteria after 6 h and 8 h (**b**). *p < 0.05. N.S., not significant; CFU, colony-forming units

In both NTHi and *S. pyogenes,* one bacterial strain (NTHi1 or *S. pyogenes*1) was used to confirm the time of cellular invasion. NTHi invaded the cells 2 h after they were attached to the cells (Fig. 1a). After 4 and 6 h, the number of bacteria invading the cells increased in a time-dependent manner (Fig. 1a). However, there was no difference between the numbers of bacteria invading the cells at 6 h and 8 h (Fig. 1a). Similarly, the number of *S. pyogenes* invading the cells increased in a time-dependent manner, and there was no difference between the numbers of bacteria invading the cells at 6 h and 8 h (Fig. 1b). Based on these results, the time to enter the cells was set to 6 h.

### MIC and *emm* genotype in *S. pyogenes*

In NTHi isolates, the MICs of GRNX, CDTR-PI, and LVFX were 8-fold lower than those of AMPC and 36-fold lower than those of CAM (Table 1). In *S. pyogenes* isolates, the MICs of GRNX, CDTR-PI, and AMPC were 8-fold lower than those of LVFX and 4-fold lower than those of CAM (Table 1). We classified 4 *S. pyogenes* strains into 4 *emm* types, as shown in Table 1.

**Table 1:** Minimum inhibitory concentrations of antibacterial agents against bacteria and *emm* genotype in *S. pyogenes*

A						
strain	MIC (μg/ml)					
	GRNX	LVFX	CAM		AMPC	CDTR-PI
NTHi 1	0.06	0.06	4		0.5	0.06
NTHi 2	0.06	0.06	4		0.5	0.06
NTHi 3	0.06	0.06	8		0.5	0.06
NTHi 4	0.06	0.06	2		0.5	0.06
NTHi 5	0.06	0.06	4		0.5	0.06
B						
strain	*emm* genotype	MIC (μg/ml)				
		GRNX	LVFX	CAM	AMPC	CDTR-PI
*S. pyogenes* 1	*emm* 89	0.06	0.5	0.25	0.06	0.06
*S. pyogenes* 2	*emm* 75	0.06	0.5	16	0.06	0.06
*S. pyogenes* 3	*emm* 11	0.12	2	0.25	0.06	0.06
*S. pyogenes* 4	*emm 28*	0.12	0.5	0.25	0.06	0.06

Genotyping of the *emm* gene encoding the M protein was performed according to the protocol presented by the Center for Disease Control and Prevention

*MIC* minimum inhibitory concentration, *NTHi* nontypeable Haemophilus influenzae, *S. pyogenes* Streptococcus pyogenes, *GRNX* garenoxacin, *LVFX* levofloxacin, *CAM* clarithromycin, *AMPC* amoxicillin, *CDTR-PI* cefditoren pivoxil

### Effects of antibacterial agents on NTHi

**Fig. 2:**
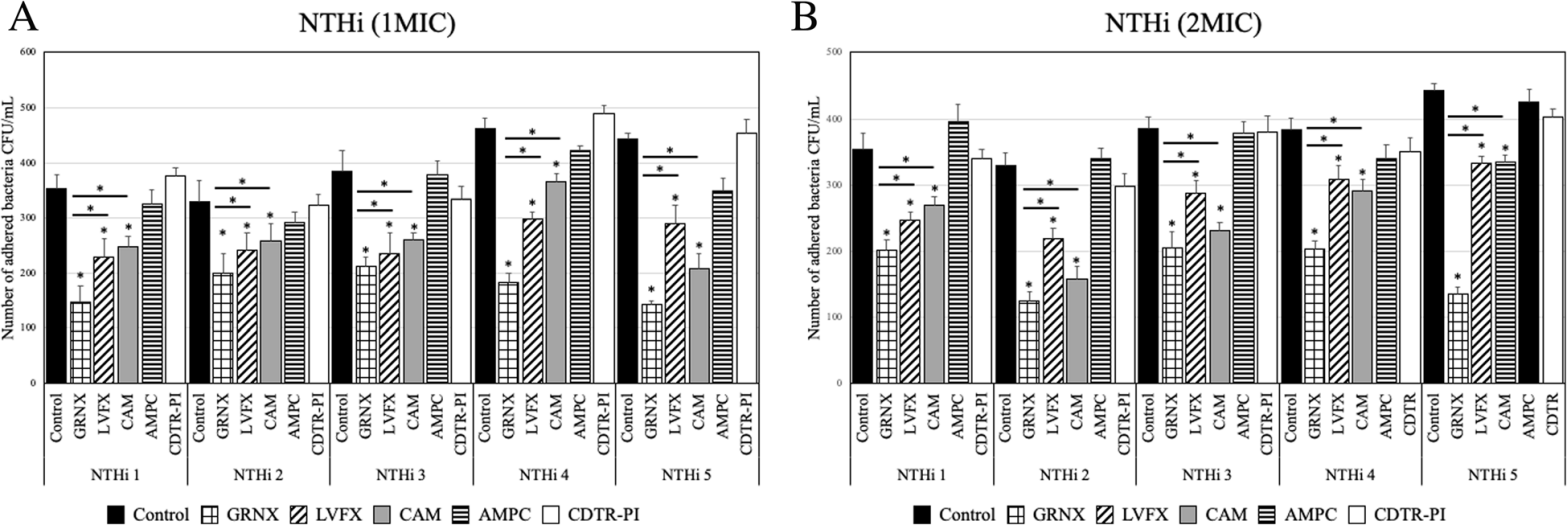
Effects of antibacterial agents on nontypeable *Haemophilus influenzae.* Cells invaded by bacteria and then treated with phosphate-buffered saline (PBS) served as the study control. A significant bactericidal effect on each NTHi strains was observed when 1 MIC of GRNX, CAM, or LVFX was used (**a**) (*p* < 0.05). However, no bactericidal effect was observed from treatment with AMPC or CDTR-PI (**a**). Similarly, treatment with 2 MIC of GRNX, CAM, or LVFX also had a significant bactericidal effect (*p* < 0.05), but when treated with AMPC or CDTR-PI, no bactericidal effect was observed (**b**). MIC, minimum inhibitory concentration; NTHi, nontypeable *Haemophilus influenzae*; GRNX, garenoxacin; LVFX, levofloxacin; CAM, clarithromycin; AMPC, amoxicillin; CDTR-PI, cefditoren pivoxil; CFU, colony-forming units. *p < 0.05

Treatment with 1 MIC of GRNX, CAM, or LVFX significantly reduced the number of cell-invaded NTHi (Fig. 2a) (*p* < 0.05). GRNX had a significantly higher bactericidal effect than CAM and LVFX (*p* < 0.05). However, no bactericidal effect was observed from treatment with AMPC or CDTR-PI (Fig. 2a).

Similarly, treatment with 2 MIC of GRNX, CAM, or LVFX also had a significant bactericidal effect (*p* < 0.05), but treatment with AMPC or CDTR-PI had no bactericidal effect (Fig. 2b). GRNX had the highest bactericidal effect (Fig. 2b).

### Effects of antibacterial agents on *S. pyogenes*

**Fig. 3:**
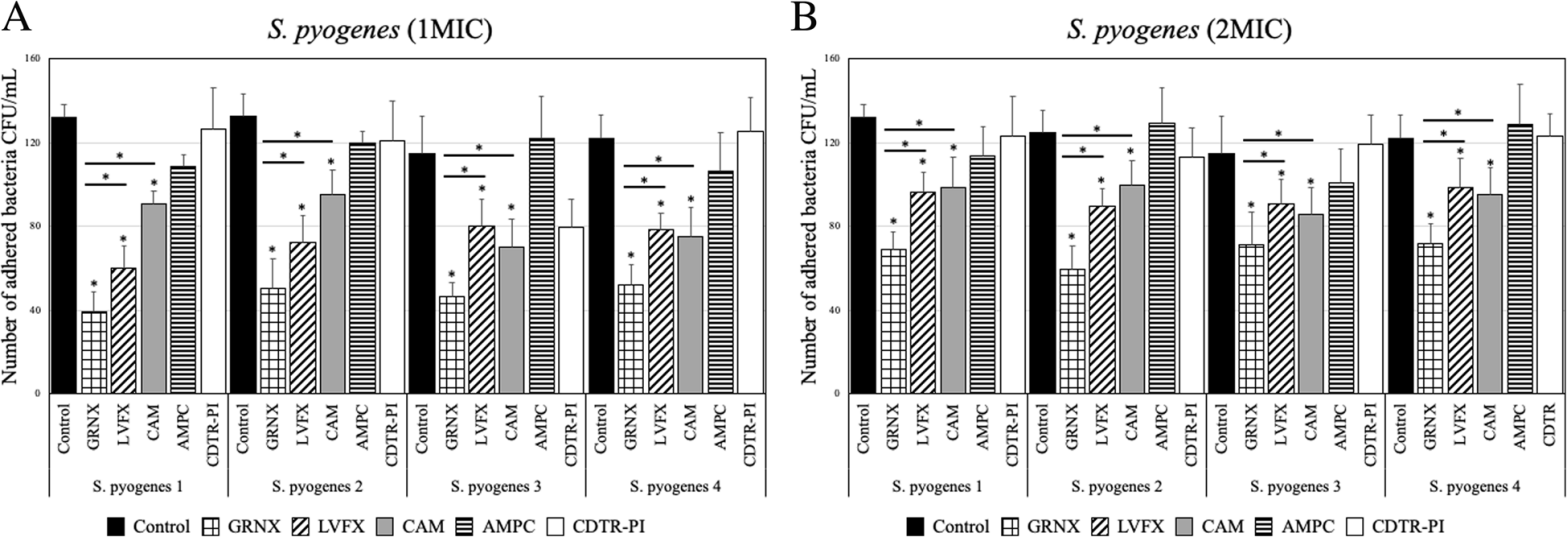
Effects of antibacterial agents on *Streptococcus pyogenes.* Cells invaded by bacteria and then treated with PBS served as the study control. When 1 MIC of GRNX, CAM, or LVFX was used, each *S. pyogenes* strain demonstrated a significant bactericidal effect (**a**) (*p* < 0.05). However, treatment with AMPC or CDTR-PI showed no bactericidal effect (**a**). Treatment with 2 MIC of GRNX, CAM, or LVFX also yielded a significant bactericidal effect (*p* < 0.05), but treatment with AMPC or CDTR-PI exhibited no bactericidal effect (**b**). MIC, minimum inhibitory concentration; *S. pyogenes*, *Streptococcus pyogenes*; GRNX, garenoxacin; LVFX, levofloxacin; CAM, clarithromycin; AMPC, amoxicillin; CDTR-PI, cefditoren pivoxil; CFU, colony-forming units. *p < 0.05

Treatment with 1 MIC of GRNX, CAM, or LVFX significantly reduced the number of cell-invaded *S. pyogenes* entering (Fig. 3a) (*p* < 0.05). GRNX had a significantly higher bactericidal effect than CAM and LVFX (Fig. 3a) (*p* < 0.05). However, no bactericidal effect was observed from treatment with AMPC or CDTR-PI (Fig. 3a).

Similarly, treatment with 2 MIC of GRNX, CAM, or LVFX also had a significant bactericidal effect (*p* < 0.05), but treatment with AMPC or CDTR-PI had no bactericidal effect (Fig. 3b). GRNX had the highest bactericidal effect (Fig. 3b).

## Discussion

This study investigated the effects of GRNX, CAM, AMPC, CDTR-PI, and LVFX on the invasion of Detroit 562 cells by NTHi and *S. pyogenes*. Results showed that NTHi and *S. pyogenes* invaded Detroit 562 cells. Nevertheless, these bacteria that invaded cells were eliminated by GRNX, CAM, and LVFX, but not by AMPC and CDTR-PI. Among them, GRNX was the most effective.

Fibronectin-binding protein (F1 protein) is mentioned as a mechanism by which *S. pyogenes* invades the cells [15]. In Japan, Ma et al. [16] reported that 77.3% of *S. pyogenes* strains possessed F1 protein. Intracellular invasion ability and biofilm formation ability are negatively correlated, and it is considered that *S. pyogenes* avoids the attack of antibacterial agents [15]. Moreover, the clinically isolated *S. pyogenes* showed serotype-specific characteristics, with the *emm*12 strain being detected most frequently and the *emm*6 strain more likely to produce biofilms [17]. In addition, the ability to invade Detroit 562 cells was significantly greater in the *emm*4, *emm*6, and *emm*75 strains than in the strains of other genotypes [17]. In this study, 4 *emm* strains classified and the number of *S. pyogenes* that invaded cells differed in each strain. However, there was no difference in the number of intracellular invasions among the 4 strains (data not shown).

In addition, phosphorylcholine is mentioned as a mechanism of intracellular invasion by NTHi, and the higher the expression level of phosphorylcholine, the more it penetrates into cells [18]. The present study showed that it takes a certain period of time for bacteria to adhere to cells and enter the cells, which becomes constant within 6 h. Yamanaka’s report that *H. influenzae* invades Detroit 562 cells supports the results of the present study [19].

Since the late 1990s, respiratory tract infections caused by antibiotic-resistant strains of *S. pneumoniae* and *H. influenzae* have increased exponentially worldwide. Penicillin-resistant *S. pneumoniae*, such as penicillin intermediately-resistant *S. pneumoniae* (PISP), penicillin-resistant *S. pneumoniae* (PRSP), and BLNAR, are particularly common in Japan [10]. In NTHi isolates, the MICs of AMPC were eightfold higher than those of GRNX, CDTR-PI, and LVFX. In *S. pyogenes* isolates, the MICs of LVFX were eightfold higher than those of GRNX, CDTR-PI, and AMPC. Quinolone disrupts the DNA replication of type II topoisomerase, thereby inhibiting bacterial growth. Moreover, type II topoisomerases include DNA gyrase and topoisomerase IV and consist of two dimers of subunit types A and B. The quinolone resistance-determining regions (QRDRs) within subunits A and B are closely related to resistance [20]. Shoji et al. [21] reported that of the 14 *S. pyogenes* strains, 12 (85.7%) had two or more mutations in QRDRs. This is considered one of the reasons why *S. pyogenes* were less sensitive to LVFX.

Invasion of cells by bacteria has been cited as a cause of repeated tonsillitis. In this study, neither AMPC nor CDTR-PI was found to have a bactericidal effect on bacteria invading the cells. It is known that β-lactam antibacterial agents have low intracellular transmissibility, and their antibacterial action is reduced against *H. influenzae* that has entered the cells [22]. Therefore, it is suggested that another antimicrobial treatment is necessary for recurrent tonsillitis.

GRNX is highly effective in the treatment of patients with upper and lower respiratory tract infections [11]. Takagi et al. [23] reported that GRNX concentrations in plasma and tissues of subjects receiving GRNX 400 mg once a day were higher than the MIC90 of major causative pathogens. The trough concentration (Cmin) in plasma was 1.92 g/mL, a level that was higher than the mutant prevention concentration, suggesting that GRNX is unlikely to induce the selection of resistant strains during treatment. The efficacy rates of GRNX in otorhinolaryngological infections were 91.3% for sinusitis, 81.8% for otitis media, 89.5% for pharyngolaryngitis, and 95.0% for tonsillitis [23]. A double-blind study was conducted comparing GRNX 400 mg once a day with LVFX 100 mg three times a day for 10 days in patients with bacterial pneumonia. The bacterial eradication rate was 100% (53/53) in the GRNX group and 87.8% (36/41) in the LVFX group. This difference in the eradication rate was statistically significant, with a 95% CI of 2.4 to 23.9% [24]. In the present study, GRNX was more effective against bacteria that invaded cells than LVFX. Moreover, LVFX was less sensitive against NTHi, and GRNX was found to be effective for recurrent tonsillitis.

The present study showed that CAM was effective against bacteria invading cells. Patel et al. [25] reported that the concentration of CAM in alveolar macrophages of healthy subjects reached a maximum of 1996 μg/mL at 4 h after administration of 500 mg of CAM. Chou et al. [26] reported that cultured human gingival fibroblasts and SCC-25 cells took up CAM via a concentrative active transport system. However, the concentration of CAM used in this study is far beyond the amount used in actual clinical practice and therefore could not be used in actual clinical practice.

Our study has some limitations. First, BLNAS and other resistant strains were not investigated. Since the number of strains of resistant bacteria is increasing, more resistant strains should be included in future studies. The second limitation pertains to the epithelial cells used. Although the use of normal human epithelial cells may be more clinically relevant, we used a pharyngeal cancer-derived cell line. Because these cells were of human origin, we consider that the results of this study were not different from those that would have been obtained with the use of normal cells.

## Conclusions

GRNX was the most effective agent against cell-invading bacteria. Administration of GRNX should be considered when the efficacy of penicillin and cephem antibiotics and of β-lactam is insufficient in daily medical practice.

## Methods

### Antibacterial agents

The following antibacterial agents were used in the study: analytical grade powders of GRNX (FUJIFILM Toyama Chemical Co., Ltd., Tokyo, Japan), CAM (Meiji Seika Pharma, Tokyo, Japan), amoxicillin (AMPC) (Wako Pure Chemical Industries), cefditoren pivoxil (CDTR-PI) (Meiji Seika Pharma, Tokyo, Japan), and LVFX (Sigma-Aldrich, Tokyo, Japan).

### Bacteria and growth conditions

We collected NTHi nasopharyngeal isolates and *S. pyogenes* oropharyngeal isolates from patients with otitis media with effusion and recurrent tonsillitis (aged 21–35 years) and patients with only recurrent tonsillitis (aged 24–42 years), respectively, at the Kagoshima University Hospital between March 2019 and December 2020. All bacteria were stored in skimmed milk with glycerol at − 80 °C until use. An aliquot of each bacterial stock was thawed and cultured overnight at 37 °C in a 5% CO_2_ incubator on chocolate II agar (Nippon Becton Dickinson Co. Ltd., Tokyo, Japan) or sheep blood agar (Nissui Pharmaceutical Co., Ltd., Tokyo, Japan) plates, as appropriate. After washing in 0.5% bovine serum albumin–phosphate-buffered saline (PBS), the bacteria were used for intracellular invasion assays. The concentrations of NTHi and *S. pyogenes* were adjusted to 1.0 × 10^8^ colony-forming units (CFU)/mL at an absorbance of 580 nm. The Institutional Review Board of Kagoshima University approved this study.

### Determination of minimum inhibitory concentration (MIC)

The susceptibility of bacteria to antibiotics was studied by the broth microdilution method, performed according to Clinical Laboratory Standards Institute guidelines [27]. The test medium was prepared using cation-adjusted Mueller Hinton broth (Eikenkagaku, Tokyo, Japan) with lysed horse blood (Nippon Biotest Laboratory, Tokyo, Japan). The quinolones evaluated were GRNX, CAM, AMPC, CDTR-PI, and LVFX. In this study, 1 and 2 MIC were used.

### Genetic characterization of *S. pyogenes*

Genotyping of the *emm* gene encoding the M protein was performed according to the protocol presented by the Center for Disease Control and Prevention (http://www.cdc.gov/ncidod/ biotech/strep/protocols.html), with minor modifications previously described [28].

### Cell culture

Detroit 562 cells (CCL-138; ATCC, Manassas, VA, USA), a human pharyngeal carcinoma epithelial cell line, were grown to confluence in minimal essential medium (Nacalai Tesque Inc., Kyoto, Japan) supplemented with 1 mM sodium pyruvate (Nacalai Tesque), 10% fetal bovine serum (Invitrogen, San Diego, CA, USA), penicillin (100 U/mL), and streptomycin (100 μg/mL; Nacalai Tesque) at 37 °C in a 5% CO_2_ incubator as previously described [29]. The cells were harvested using trypsin (final concentration, 0.02%) and ethylenediaminetetraacetic acid (EDTA; final concentration, 0.02%; Nacalai Tesque) and seeded at a density of 2 × 10^4^ viable cells per well in a 96-well BD Falcon tissue culture plate with a low-evaporation lid (BD Biosciences, Franklin Lakes, NJ, USA). The plates were used when > 90% confluence was observed following overnight incubation.

### Intracellular invasion assay

One hundred microliters each of the NTHi and *S. pyogenes* strains (1.0 × 10^8^ CFU/mL) were added to Detroit 562 cells cultured in a 96-well plate and allowed to adhere at 37 °C in a 5% CO_2_ incubator for 6 h. Each well was then treated with gentamicin (200 μg/mL) at 37 °C in a 5% CO_2_ incubator for 1 h. After washing five times with 200 μL of PBS, the cells were treated with 100 μL of each antibacterial agent at 37 °C in a 5% CO_2_ incubator for 6 h. Controls were treated with PBS without antibacterial treatment. After washing five times with 200 μL of PBS, the cells were treated with 100 μL of saponin at 37 °C in a 5% CO_2_ incubator for 15 min. Further, 100 μL of the samples from each well was plated on chocolate II agar plates or sheep blood agar and cultured overnight, and the number of colonies formed was counted as described previously [18].

### Statistical analysis

All statistical data were analyzed using SPSS for Windows software (version 22.0; IBM Corp., Armonk, New York, USA), and the values were presented as mean ± standard deviation. Furthermore, we used unpaired one-way analysis of variance with Tukey’s method for statistical data analysis. Differences showing *p* < 0.05 indicated statistical significance.

## Data Availability

The datasets used and analyzed during the current study are available from the corresponding author on reasonable request.

## Abbreviations

*S. pyogenes*: *Streptococcus pyogenes*
NTHi: Nontypeable *Haemophilus influenzae*
GRNX: Garenoxacin
CAM: Clarithromycin
AMPC: Amoxicillin
CDTR-PI: Cefditoren pivoxil
LVFX: Levofloxacin
BLNAR: Beta-lactamase-negative ampicillin-resistant *H. influenzae*
CAP: Community-acquired pneumonia
PBS: Phosphate-buffered saline
CFU: Colony-forming units
MIC: Minimum inhibitory concentration
F1 protein: Fibronectin-binding protein
PISP: Penicillin-intermediate *S. pneumoniae*
PRSP: Penicillin-resistant *S. pneumoniae*
QRDRs: Quinolone resistance-determining regions
